# From Mesoscopic Functionalization of Silk Fibroin to Smart Fiber Devices for Textile Electronics and Photonics

**DOI:** 10.1002/advs.202103981

**Published:** 2021-11-21

**Authors:** Ronghui Wu, Liyun Ma, Xiang Yang Liu

**Affiliations:** ^1^ College of Ocean and Earth Sciences State Key Laboratory of Marine Environmental Science (MEL) Xiamen 361005 P. R. China

**Keywords:** electronics, fiber devices, mesoscopic structures, photonics, silk fibroin

## Abstract

*Bombyx mori* silk fibers exhibit significant potential for applications in smart textiles, such as fiber sensors, fiber actuators, optical fibers, and energy harvester. Silk fibroin (SF) from *B. mori* silkworm fibers can be reconstructed/functionalized at the mesoscopic scale during refolding from the solution state into fibers. This facilitates the mesoscopic functionalization by engaging functional seeds in the refolding of unfolded SF molecules. In particular, SF solutions can be self‐assembled into regenerated fiber devices by artificial spinning technologies, such as wet spinning, dry spinning, microfluidic spinning, electrospinning, and direct writing. Meso‐functionalization manipulates the SF property from the mesoscopic scale, transforming the original silk fibers into smart fiber devices with smart functionalities, such as sensors, actuators, optical fibers, luminous fibers, and energy harvesters. In this review, the progress of mesoscopic structural construction from SF materials to fiber electronics/photonics is comprehensively summarized, along with the spinning technologies and fiber structure characterization methods. The applications, prospects, and challenges of smart silk fibers in textile devices for wearable personalized healthcare, self‐propelled exoskeletons, optical and luminous fibers, and sustainable energy harvesters are also discussed.

## Graphical abstract

This review summarizes the progress of mesoscopic structural construction from silk fibroin materials to fiber devices. The significance of meso‐functionalization of the mesoscopic hieratical structure of silk fibers in fiber electronics/photonics are highlighted. Applications, prospects, and challenges of functional silk fibers in textile devices for wearable personalized healthcare, self‐propelled exoskeletons, optical and luminous fibers, and sustainable energy harvesters are discussed.

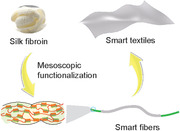

## 1 Introduction

**Figure 1:**
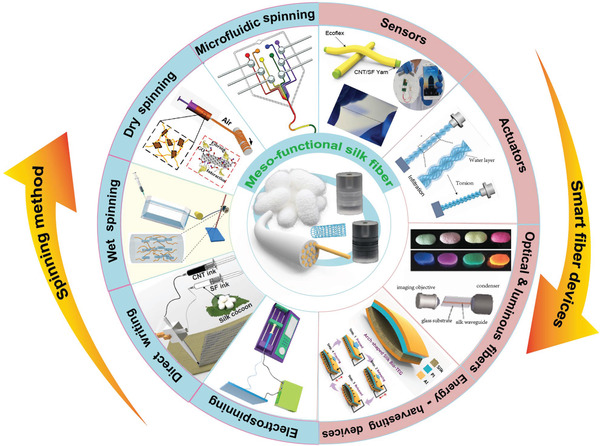
Schematic of the development of various smart fiber devices from meso‐functionalized silk fiber based on different spinning methods. Meso‐functional silk fiber: Reproduced with permission.^[^ ^35^ ^]^ Copyright 2020, Wiley‐VCH. Microfluidic spinning: Reproduced with permission.^[^ ^39^ ^]^ Copyright 2011, Springer Nature. Dry spinning: Reproduced with permission.^[^ ^40^ ^]^ Copyright 2016, American Chemical Society. Wet spinning: Reproduced with permission.^[^ ^35^ ^]^ Copyright 2020, Wiley‐VCH. Direct writing: Reproduced with permission.^[^ ^41^ ^]^ Copyright 2019, Cell Press. Sensor: Reproduced with permission.^[^ ^35^ ^]^ Copyright 2020, Wiley‐VCH. Actuator: Reproduced under the terms of the CC‐BY license.^[^ ^42^ ^]^ Copyright 2020, the Authors. Published by Wiley‐VCH. Luminous fiber: Reproduced with permission.^[^ ^43^ ^]^ Copyright 2011, Wiley‐VCH. Optical fiber: Reproduced with permission.^[^ ^44^ ^]^ Copyright 2009, Wiley‐VCH. Energy harvester devices: Reproduced with permission.^[^ ^45^ ^]^ Copyright 2016, Wiley‐VCH.

Fibrous or textile electronics and photonics are devices integrated in flexible yarns or textiles that perform smart sensing or logic functions.^[^ ^1^, ^2^ ^]^ This special type of electronic or photonic elements significantly impact smart clothes,^[^ ^3^ ^]^ which are applicable for movable energy harvesting,^[^ ^4^, ^5^, ^6^, ^7^, ^8^ ^]^ remote diagnosis,^[^ ^3^, ^9^ ^]^ personalized medical healthcare,^[^ ^10^, ^11^, ^12^, ^13^ ^]^ long‐time motion tracking, human–machine interaction,^[^ ^14^, ^15^, ^16^ ^]^ and wearable display.^[^ ^17^ ^]^ Silk fibers from silkworms are one of the most important natural fibers for applications in smart and wearable electronics (**Figure** **1**).^[^ ^18^, ^19^ ^]^ *Bombyx mori* silk fibers are world‐renowned, fine continuous protein fibers. They are regarded as the queen of fibers, and have been widely used owing to their abundant natural sources, robust mechanical properties, biocompatibility, biodegradability, heat conductivity, and wide optical window.^[^ ^18^, ^19^, ^20^, ^21^ ^]^ Consequently, *B. mori* silkworm silk fibers are potential candidates for the fabrication of various smart textile electronics. Nevertheless, the properties of natural *B. mori* silkworm silk fibers cannot completely satisfy the requirements of smart textiles, such as sensitivity to different external stimuli, responsiveness, and self‐adaptability to the environment (such as humidity and temperature). Therefore, enhancing the functionalities in a controlled and consistent manner via structural modifications will extend the applications of natural *B. mori* silkworm silk fibers. *B. mori* silkworm fibers contains two protein materials, i.e., silk fibroin (SF) and sericin protein. SF provides most characteristics and performances for application, while sericin proteins are hydrophilic and need be removed before using. Using artificial spinning technologies, such as wet spinning, dry spinning, microfluidic spinning,^[^ ^22^, ^23^ ^]^ electrospinning, and direct writing, smart silk fiber devices with specific functionalities, such as sensors,^[^ ^3^, ^20^, ^24^ ^]^ actuators,^[^ ^25^ ^]^ optical fibers,^[^ ^26^ ^]^ luminous fibers,^[^ ^27^ ^]^ and energy harvesters^[^ ^28^, ^29^, ^30^ ^]^ can be developed, as shown in Figure 1.

**Figure 2:**
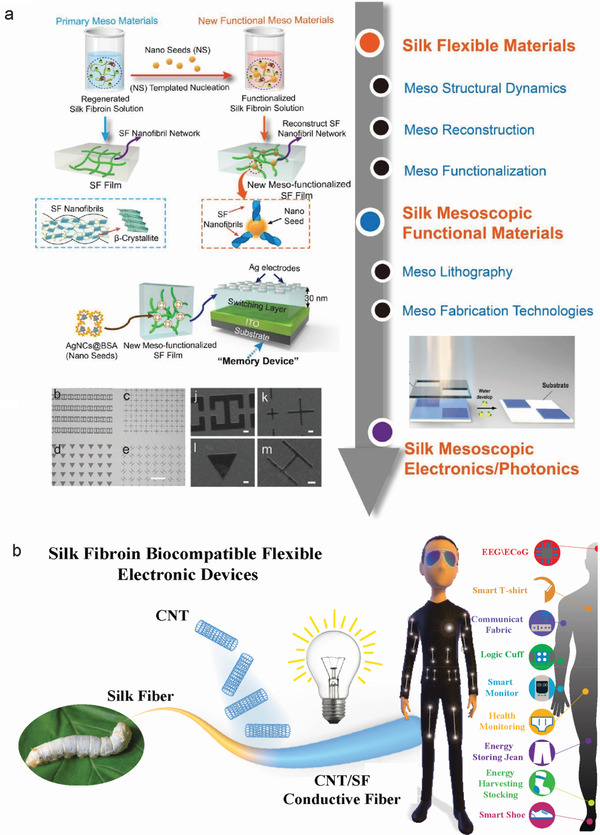
a) Strategy for the conversion of flexible functional silk materials to meso‐functionalized silk devices. Reproduced with permission.^[^ ^18^ ^]^ Copyright 2021, Wiley‐VCH. Silk mesoscopic functional materials: Reproduced with permission.^[^ ^46^ ^]^ Copyright 2019, Wiley‐VCH. Meso lithography: Reproduced with permission.^[^ ^47^ ^]^ Copyright 2019, Wiley‐VCH. b) SF biocompatible flexible electronic devices fabricated through a meso reconstruction template comprising carbon nanotube. They have many electronic applications including humidity sensors, human health monitoring, and human‐environment communications. Reproduced with permission.^[^ ^35^ ^]^ Copyright 2020, Wiley‐VCH.

For soft materials, including *B. mori* silkworm SF materials, the macroscopic performance is mainly affected by the structure at the mesoscale, instead of that at the atomic or nanoscale.^[^ ^18^, ^19^, ^31^, ^32^ ^]^ Meso‐functionalization, which endows the materials with specialized or designed functionalization from mesoscopic of view, provides a crucial approach for endowing the soft matter with novel functionalities.^[^ ^33^, ^34^ ^]^ More specifically, meso‐functionalization covers the following three approaches: i) meso‐reconstruction, ii) meso‐doping, and iii) meso‐hybridization. Similarly, for polymer fibers, functionalization based on the meso‐structure modification of spinnable polymers can endow the fibrous devices with additional capabilities, such as pressure/humidity responsiveness,^[^ ^35^, ^36^ ^]^ enhanced optical performance and energy harvesting.^[^ ^37^, ^38^ ^]^ In addition, unlike 2D or 3D silk materials, such as films or sponges, 1D orderly assembly on the mesoscopic scale, which affects the ultimate performance of the fiber, requires a complex spinning technology that can facilitate nanofibrils alignment.^[^ ^21^ ^]^Therefore, mesoscopic engineering for functionalization of SF material and spinning technology are the two key steps in engineering smart silk fiber devices,^[^ ^35^ ^]^ as shown in **Figure** **2**a. For example, mesoscopic doping by a carbon nanotube template can be adopted to fabricate a novel biocompatible silk meso‐fiber for various electronic applications, including humidity sensors, remote respiratory detecting, and disease diagnosis,^[^ ^35^ ^]^ as shown in Figure 2b. Although several reports have confirmed the importance of the meso‐structure in the design of smart silk devices, there have been very few reports that summarize the relationship between the SF meso‐structure and *B. mori* silkworm silk fiber devices.

In this review, we provide a systematic overview of the recent development in smart silk fiber devices using mesoscopic functionalization strategies. First, we introduce the different levels of hierarchical structures of the SF material from the mesoscopic scale (Section 2.1), followed by introduction of several characterization techniques for fiber structure analysis (Section 2.2), and the explanation of the meso‐reconstruction and refolding of SF controlled by *β*‐crystallite nucleation (Section 2.3). Then, we introduce artificial spinning methods used to convert the SF material from a solution into 1D fibers and discuss the SF assembly during the process (Section 3). The spinning conditions associated with the alignment and crystallization of SF molecules are also discussed. In the last section, we summarize the recent developments in functionalized or enhanced SF materials that enable smart *B. mori* silkworm silk fiber devices, including fiber sensors and actuators, optical fibers, florescent fibers, and energy‐harvesting fiber‐based devices (Section 4). Moreover, the potential applications of and the challenges associated with smart *B. mori* silkworm silk fibers in wearable personalized healthcare, self‐propelled exoskeletons, and sustainable energy harvesters are highlighted. This review ranges from the fundamental hierarchical network structures of silk materials to the fibrous smart devices that constitute the basic elements of textile electronics and photonics, and summarizes the research progress on the silk fiber electronics and photonics, which will assist in the comprehensive investigation of smart *B. mori* silkworm fibers.

## 2 Hierarchical Meso‐Structure and Meso‐Functionalization of Natural SF Materials

### 2.1 Hierarchical Meso‐Structure of Natural SF Materials

**Figure 3:**
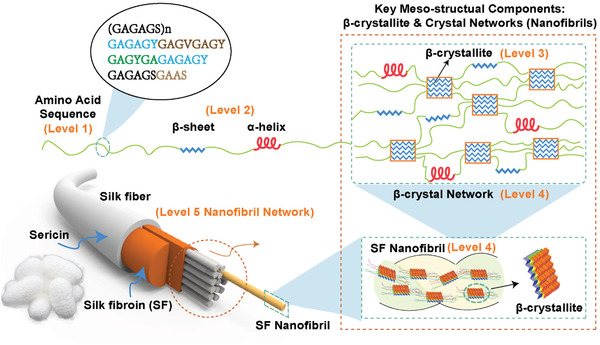
Five‐level hieratical structure of SF materials. i) Primary structure (level 1): amino acid sequence; ii) Secondary structure (Level 2): *α*‐helices and *β*‐sheets; iii) Tertiary structure (Level 3): *β*‐crystallites; iv) Quaternary structure (Level 4), *β*‐crystal networks, in which *β*‐crystallites are connected to one another by amorphous chains (*α*‐helices and/or random coils); v) Quinary structure (Level 5): Nanofibrils’ bundle/network. Reproduced with permission.^[^ ^35^ ^]^ Copyright 2020, Wiley‐VCH.

*B. mori* silkworm silk fibers exhibit excellent mechanical properties, which correlate with their multilevel network structures,^[^ ^19^ ^]^ which can be divided into five levels (**Figure** **3**): amino acid sequences (primary structure), *β*‐sheets and *α*‐helices (secondary structure), *β*‐crystallites (tertiary structure), crystal network (nanofibrils, quaternary structure), and nanofibril network (quinary level structure).^[^ ^35^ ^]^

#### 2.1.1 Primary Structure of B. mori Silk Fibers

The primary structure of silk protein materials refers to the amino acid sequence. *B. mori* silkworm silk fibers comprise SF and sericin. The primary mechanical properties of silk fibers are attributed to the SF. Consequently, the discussion in this section is based on SF. SF can be further divided into two categories: heavy chain proteins (H) and light chain proteins (L). The heavy and light chains form H—L complexes via the formation of disulfide bonds.^[^ ^48^ ^]^ Both proteins contain amino acids: glycine (G), alanine (A), serine (S), and tyrosine (Y) are the main components of heavy chain proteins.^[^ ^49^ ^]^ The amino acids in heavy chain proteins are distributed in hydrophobic and hydrophilic regions. The hydrophobic region mainly comprises glycine “X” (X refers to other types of amino acids like alanine, serine, and tyrosine) in irregular repeating sequences such as GAGAGS and GAGAGY. Different substructure regions play different roles. For example, the (GAGAGS)*_n_* repeat sequence is the main unit of *β*‐sheets.^[^ ^50^, ^51^, ^52^ ^]^ In short, differences in the amino acid sequence directly affect the macro‐mechanical properties of silk fibers.

#### 2.1.2 Secondary Structure of B. mori Silk Fibers

The secondary structure of proteins is a relatively stable structure formed by hydrogen bonding of the main chain in the protein molecule. *α*‐helices and *β*‐sheets are the most common secondary structures in silk fibers. There are numerous hydrogen bonds in the *α*‐helice structure, which is more stable than random coils. The direction of the hydrogen bonds is generally parallel to that of the *α*‐helice. In the *β*‐sheet structure, hydrogen bonds are located between adjacent *β*‐strands (GAAS tetrapeptides),^[^ ^53^ ^]^ which limits the size of the *β*‐crystallites. Although a single hydrogen bond is much weaker than a covalent bond, the abundant hydrogen bonds in the *β*‐sheet structure ensures its stability. The *β*‐sheet structure is more stable than random coils and *α*‐helices.^[^ ^54^ ^]^ When the silk protein material has a high *β*‐sheet content, it is not easily hydrolyzed and is more rigid. However, when the content of the *α*‐helice structure is higher, the silk protein material exhibits better elasticity.

#### 2.1.3 Tertiary Structure of B. mori Silk Fibers

The tertiary structures of SF materials are intermolecular *β*‐crystallites^[^ ^31^, ^55^, ^56^ ^]^ formed by stacking numerous *β*‐sheets of different molecules through various interactions. In *β*‐crystallites, the interaction that stabilizes its structure is termed the “crystallization force,” which includes hydrogen bonds, hydrophobic interactions, van der Waals forces, and other forces. A stronger crystallization force corresponds to a more stable crystallite structure.^[^ ^31^, ^53^, ^55^ ^]^ The stacking and interaction of *β*‐sheets influence the crystallite structure. In addition, the size of the *β*‐crystallites impacts their stability and the mechanical performance of the fiber, which can be characterized by X‐ray diffraction. The approximate dimensions of the *β*‐crystallites are a = 9.2 Å, b = 9.4 Å, and c = 6.94 Å, where a is the stacking direction of *β*‐sheets (intersheet direction), b is the direction of the *β*‐sheet perpendicular to the chain axis (backbone‐backbone hydrogen bonding direction, or interchain direction), and c is the strand axis direction.^[^ ^19^, ^57^ ^]^ Wu et al.^[^ ^58^ ^]^ reported the mechanism of *β*‐sheet splitting (within *β*‐crystallites). During the stretching process of *B. mori* silk fibers, the crystallite splitting is due to destruction of the internal *β*‐sheet interactions. Destruction of the *β*‐sheets results from breaking of the hydrogen bonds between adjacent *β*‐strands inside the *β*‐sheet. Because the direction of the *β*‐chains in the *β*‐sheets and *β*‐crystallites is consistent with the axial direction of the silk fiber, the stability of the crystallites is stronger when the crystallite size in this direction (i.e., the c‐direction) is larger, within a certain size range.^[^ ^31^ ^]^ The influence of the crystallite size in the b‐direction on the crystallite stability is often reflected in the influence on the strength, stiffness, and toughness of the fiber.^[^ ^59^ ^]^ Naturally, there are more defects or mismatches in large crystallites than in small crystallites due to the mismatch between crystal nuclei, which reduces the mechanical stability of large crystallites.

*B. mori* silk fiber exhibits excellent mechanical properties due to *β*‐crystallites and crystal network. Specifically, *β*‐crystallites are composed of several adjacent *β*‐sheets from different molecules, in which hydrogen bonds and hydrophobic interactions/van der Waals interactions play a key role. In this respect, these interactions can also be referred to as crystal binding interactions/forces. Although hydrogen bonds and hydrophobic interactions are much weaker than covalent bonds. However, if the binding entity is in a crystalline state, the binding force can be significantly enhanced. Unlike the amorphous state where each noncovalent bond/interaction can be broken separately, in the crystalline bonding state, the minimum breaking force of the bonded crystallites is equivalent to the simultaneous breaking of all noncovalent bonding interactions under critical conditions. This is because if the crystallite size is smaller than the critical size, the crystallite becomes unstable. This produces the collectiveness of noncovalent bonds within the volume, showing stronger bonding. In this case, although a single noncovalent interaction is weak, a combination of noncovalent interactions can lead to a strong bonding situation. This encourages strong connections and stable bonding points in the network of silk materials. In this regard, *β*‐crystals play a key role in stabilizing the microstructure of silk protein materials. Therefore, without *β*‐crystals, the silk protein material is very unstable and highly water‐soluble.

#### 2.1.4 Quaternary Structure of B. mori Silk Fibers

The quaternary structure of *B. mori* silk fibers comprises fishnet‐like crystallite networks and nanofibrils, which are detailed below.^[^ ^31^, ^55^, ^60^, ^61^ ^]^ As a structurally stable unit, the above‐mentioned *β*‐crystallites (tertiary structure) can impart strong mechanical properties to SF fibers, but they are not sufficient to impart super‐strong mechanical properties. When numerous *β*‐crystallites are connected to form a network structure (quaternary structure), i.e., nanofibrils (crystal network), the silk protein can be endowed with sufficient strength. Liu et al. observed the existence of nanofibrils in different silk protein materials (such as fibers, silk protein hydrogels, films, and scaffolds). The interior of the nanofibril is essentially a “nanofishnet” crystallite network formed by connecting numerous crystallites by amorphous chains. These crystallites can be used as nodes in the nanofishnet owing to their high stability.^[^ ^55^ ^]^ The nanofibrils endure the most pulling force during stretching.

Owing to the shearing force, the *β*‐sheets are oriented parallel to the shearing force. When the orientation of the crystallites formed by the *β*‐sheets is consistent with the direction of the force applied to the silk protein material, the hydrogen bonds can be fully utilized to stabilize the crystallites. A spinning solution forms fibers under the action of the fluid shearing force. Based on the above discussion, the developed crystalline orientation of the *β*‐sheets improves the macroscopic mechanical properties of the fibers. In addition, Liu et al. explored the phenomena of strain strengthening and weakening by analyzing the structure of silk fibers during tension.^[^ ^54^ ^]^

#### 2.1.5 Quinary Structure of B. mori Silk Fibers

Each individual nanofibril in the quaternary structure is essentially a *β*‐crystallite network. Silk proteins comprise nanofibril networks formed by the interaction of numerous nanofibrils (quinary structure). Morphological characterization using atomic force microscopy (AFM) revealed that the nanofibrils are disordered in silk protein hydrogels. Because the silk fiber is subjected to a strong shearing effect during the spinning process, the internal nanofibrils (with unique helical characteristics) are closely bound and almost parallel, thus making it difficult for closely adjacent nanofibrils in the silk fiber to slide when stretched by an external force. In other words, the arrangement and unique morphology of the nanofibrils endow the nanofibrils with strong interaction and provide silk fibers with good mechanical properties.

#### 2.1.6 Meso‐Structural Models of B. mori Silk Fibers

**Figure 4:**
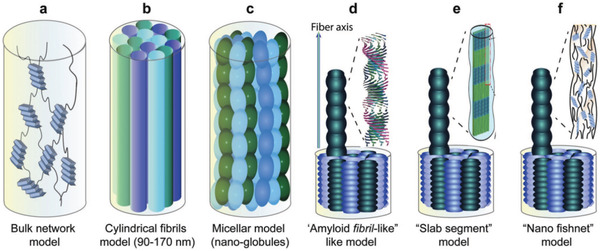
Six models showing the possible mesoscopic structures of natural *B. mori* silk fibers. a) Bulk network model, b) cylindrical fibril model, c) micellar model, d) amyloid fibril‐like model, e) slab segment model, and f) nanofishnet model. a–f) Reproduced with permission.^[^ ^19^ ^]^ Copyright 2019, Wiley‐VCH.

The excellent macroscopic performance of *B. mori* silk fibers primarily depends on the unique hierarchical structure of the fiber. In recent years, various models of this structure have been proposed (**Figure** **4**). The first is the semicrystallite (bulk network) model.^[^ ^62^, ^63^ ^]^ In this model, *B. mori* silk fibers are a type of composite material in which crystalline regions are embedded in the amorphous regions. The crystalline regions endow the silk fibers with strength, whereas the noncrystalline regions provide fiber elasticity. However, the model is too simplistic and only considers the nanoscale molecular structure of the silk fibers. The second model is the cylindrical fibril model,^[^ ^56^ ^]^ which is formed by observing the fibrils (diameter: 90–170 nm) on the surface of the silk fibers. In this model, the interaction between adjacent fibrils can effectively disperse the stress, thus rationalizing the high strength of the silk fibers. The third model is the micellar model,^[^ ^56^ ^]^ which assumes that micelles (diameter: ≈100 nm) coalesce and elongate to form the basic microstructural units of silk fibers rather than fibrils. All three models have limitations, and with the improvement in characterization methods and the continuous progress of research, amyloid‐like fibril, slab segment, and nanofishnet models have been proposed. The possibility of the amyloid fiber‐like and slab‐segment models was eliminated by the latest nanofishnet model.^[^ ^60^ ^]^ As the nodes of the nanofishnet, the *β*‐crystallites can strengthen silk fibers by sharing external forces in the optimized network.

### 2.2 Meso‐Structural Characterization of *B. mori* Silk Fibers

#### 2.2.1 Fourier Transform Infrared Spectroscopy (FTIR)

As a characterization method, FTIR is one of the most extensively used techniques for researching on protein secondary structures because it can be used to determine molecular vibration and rotation information.^[^ ^35^ ^]^ Because the vibration frequencies of different bonds are distinct, the corresponding spectra can be employed to identify the molecular structures. In particular, FTIR is often used to acquire the secondary structures of silk proteins in the solid state. According to the characteristics of infrared spectroscopy and the structure of SF, amide I vibration bands are generally used for analysis because it is the only amide vibrational band that relies on the secondary structure of the protein backbone. Further, it has strong overlapping components corresponding to different secondary structures, including random coils, *α*‐helices, *β*‐turns, and *β*‐sheets.

**Figure 5:**
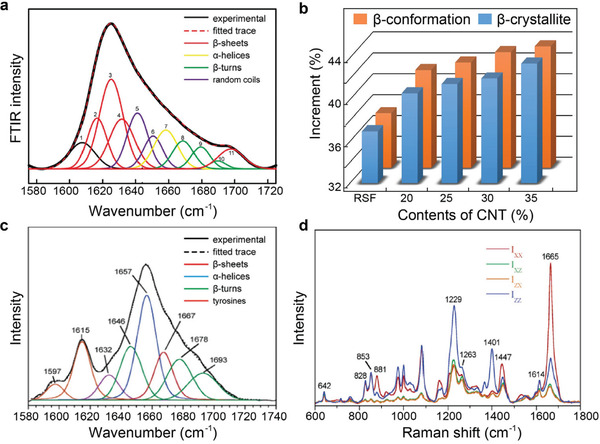
Characterization methods for the secondary structures of silk materials. a) FTIR spectra deconvolution and peak assignment. Reproduced with permission.^[^ ^19^ ^]^ Copyright 2019, Wiley‐VCH. b) Information regarding *β*‐crystallites and *β*‐conformations obtained by FTIR and WAXD. Reproduced with permission.^[^ ^35^ ^]^ Copyright 2020, Wiley‐VCH. c) Raman band decomposition of the amide I region of the isotropic spectra for SF fibers. Reproduced with permission.^[^ ^65^ ^]^ Copyright 2006, American Chemistry Society. d) Polarized spectra of *B. mori* SF fibers. Reproduced with permission.^[^ ^66^ ^]^ Copyright 2004, American Chemistry Society.

The peak deconvolution method^[^ ^64^ ^]^ can be used to fit the amide I band with various Gaussian peaks representing different secondary structures. The content of each secondary structure can be measured by the area ratio under the corresponding peak in these regions (**Figure** **5**a). Recently, time‐resolved FTIR has also been extended to continuously monitor the kinetics of conformational transitions caused by various environmental factors (e.g., metallic ions, PH, and organic solvents).^[^ ^19^, ^65^ ^]^ In addition, FTIR was used to analyze the influence of additional materials on the conformational transition of formed SF fibers. As shown in Figure 5b, Liu et al. used the above FTIR quantitative analysis method to reveal that the additional material promotes the nucleation of *β*‐crystallites and the formation of SF nanofibril networks in the meso‐reconstructed hybrid fiber. Thus, the content of *β*‐conformations (*β*‐sheets and *β*‐crystallites) gradually increases as the amount of nucleation seeds, i.e. carbon nanotubes (CNTs) increases (Figure 5b).^[^ ^35^ ^]^

#### 2.2.2 Raman Spectroscopy

Similar to FTIR analysis, Raman spectroscopy can be used to quantitatively analyze the secondary structure content in silk materials.^[^ ^35^ ^]^ The data obtained from FTIR and Raman spectroscopy are complementary. Raman spectroscopy uses scattering spectra to obtain information on the vibration and rotation of molecules and can be used for molecular structure analysis (Figure 5c).^[^ ^19^ ^]^

Raman spectroscopy techniques have improved over time. By reducing the size of the incident laser beam, data signals can be collected from very small samples, allowing effective Raman analysis of a single silk fiber. The frequency of some bands of *B. mori* silk fibers shift under tensile stress or strain, which indicates a conformational change in the molecule. Therefore, this technology can be used to detect the in situ transformation of the secondary structures of silk fibers under mechanical stretching. Notably, the mechanical properties of the fiber along the axial direction primarily depend on the orientation of the multilayer structure. Raman spectroscopy can also be used to study the orientation of silk protein molecular chains. The orientation of *β*‐sheets along the fiber axis can be analyzed using the characteristic band positions of amide I and amide III in the Raman spectra obtained from the fiber axis parallel and vertical to the laser polarization direction (Figure 5d).^[^ ^67^ ^]^

#### 2.2.3 Circular Dichroism （CD） Spectroscopy

CD spectroscopy is more suitable than FTIR and Raman spectroscopy for characterizing the secondary structure content of proteins in solution. Because the chirality of protein molecules is completely dependent on the secondary conformation, and the CD spectrum is particularly sensitive to chirality, secondary structure information can be obtained from CD measurements.^[^ ^68^ ^]^ The conformational transitions of SF in both organic and aqueous solvents can be studied and the conformational transition kinetics can be measured by CD spectroscopy. Although CD spectroscopy can be used to detect the secondary structure of SF solutions, the concentration of the protein solution must be appropriate. In general, only very dilute SF solutions are suitable for CD analysis.

#### 2.2.4 Wide‐Angle X‐Ray Diffraction (WAXD) and Small‐Angle Angle X‐Ray Scattering (SAXS)

As mentioned previously, the third, fourth, and fifth levels of the structures of SF materials are directly associated with *β*‐crystallites and crystal networks. The characteristics of the structures, including the size, density, and ordering parameters, can be determined by various techniques, including WAXD and SAXS.^[^ ^69^, ^70^ ^]^ Because X‐ray diffraction measures crystals in real space, it causes diffraction in reciprocal space, and the orientation and intensity of the diffraction patterns can be mapped to the structures of the crystals. WAXD (or wide‐angle X‐ray scattering, WAXS) and SAXS can be applied to determine the crystal size.^[^ ^71^ ^]^ WAXS is mostly used to examine the size and orientation of the crystals and the crystallinity in the crystal networks of silk materials. SAXS technology is mostly used to determine the intercrystalline distance in the crystal network of silk fibers.^[^ ^71^ ^]^

**Figure 6:**
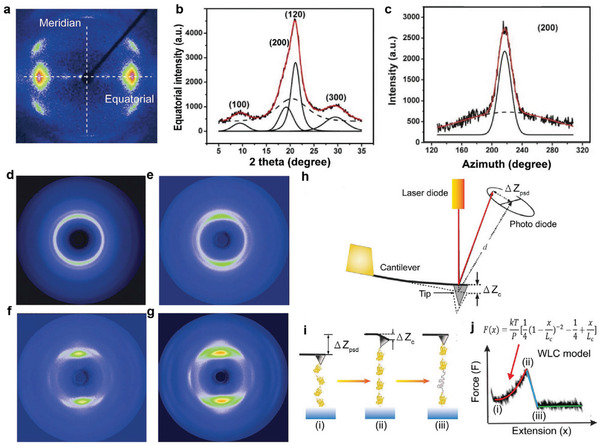
X‐ray diffraction and AFM analysis of the structural information of SF materials. a) Representative scattering pattern of a silkworm silk fiber. b) Equatorial intensity as a function of diffraction angle (2*θ*). c) Intensity as a function of azimuth angle at the radial position of the equatorial (200) peak of silkworm silk fibers. a–c) Reproduced with permission.^[^ ^19^ ^]^ Copyright 2019, Wiley‐VCH. Synchrotron radiation 2D WAXD patterns of RSF/CNT composite fibers with different draw‐down ratios: d) 1, e) 2, f) 6, and g) 9 times. d‐g) Reproduced with permission.^[^ ^72^ ^]^ Copyright 2015, the Royal Society of Chemistry. h) Schematic illustration of the AFM testing. The deflection and position of cantilever were used to measure the force and the strain, respectively. i) Unfolding of a protein domain (or *β*‐crystallite in the case of SF molecular) by the external force. The protein begins to unravel when the force is applied. From states i) to ii), the distance between the substrate and cantilever increases along with the protein elongates. The entropy reduction creates a restoring force and bends the cantilever. In the state of iii), protein contour length increases, the force on the cantilever is returned to near zero. Further extension generates a force on the cantilever and goes to state i) again. j) The entropic elasticity of proteins can be explained by the worm‐like chain (WLC) model (inset) of polymer elasticity. This equation predicts the entropic restoring force (F) generated during protein elongation (x) in terms of its duration (P) and its contour length (Lc). The serrated peak pattern, which is related to force‐elongation relationship, corresponds to the continuous unraveling of individual domains of modular proteins as shown here. The peak numbers correspond to the domain numbers.h–j) Reproduced with permission.^[^ ^19^ ^]^ Copyright 2019, Wiley‐VCH.

For crystallinity size measurement, the equatorial and meridian integrals of the WAXS pattern (**Figure** **6**a) provide a 1D profile of scattering intensity as a function of the 2*θ* angle (Figure 6b). Then, this profile can be deconvolved into a halo (corresponding to amorphous regions) and some peaks (corresponding to crystalline domains). For silk fibers, the equatorial data are deconvolved into an amorphous halo and four crystal peaks related to the (100), (200), (120), and (300) Bragg reflections. Moreover, the meridian data are deconvoluted into an amorphous halo and two additional crystal peaks corresponding to (002) and (102). Crystallinity is determined by the ratio of the area below the crystalline peaks in the equatorial data (i.e., the (100), (200), (120), and (300) peaks) to that of the complete reflection pattern.^[^ ^19^ ^]^ Using Scherrer's formula and the above information, the crystalline size can be obtained. The crystallite orientation can be determined by integrating the WAXS intensity as a function of the azimuth angle at the radial positions of the equatorial (120) and (200) peaks (Figure 6c). For example, Shao et al. demonstrated that artificial regenerated silk fibroin (RSF) yarns exhibit clear diffraction patterns, indicating that these yarns have a certain degree of crystallinity.^[^ ^72^ ^]^ Moreover, the diffraction pattern of the fiber in the meridian direction becomes clearer with increasing draw ratio, which indicates an increase in the crystallinity of the fiber (Figure 6d–g).

#### 2.2.5 AFM Force Spectroscopy for Verification of the Mesoscopic Crystal Network Structure of SF Fibrils

AFM force spectroscopy is a single‐molecule characterization technique that is often used to measure structural changes in the unfolding or refolding process of proteins or DNA. Simply, when the sample was approached and snapped by the AFM cantilever, the machine was used to stretch the sample. By recording the degree of deflection of the cantilever versus piezo movement, the detailed mechanical information of the sample can be obtained, and the internal nanostructure can be inferred. For example, AFM‐based force spectroscopy can be used to assess different mechanical properties, including the viscosity, Young's modulus, and stiffness. Recently, it has also been used to explore the topological structure of nano‐*β*‐crystallite networks within silk protein materials. By studying the probability that *β*‐sheets are pulled or that *β*‐strands are unzipped as the *β*‐crystallite is torn by tension, it is possible to understand how these crystallites interact with each another to form crystal networks (Figure 6h–j).^[^ ^19^ ^]^

#### 2.2.6 Imaging Techniques

**Figure 7:**
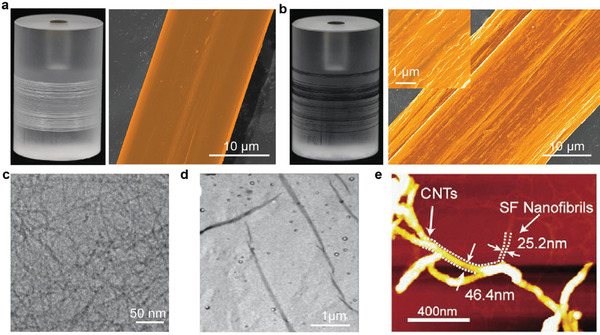
Image characterization of the morphology of SF materials. Optical and SEM images of typical surface morphologies of a) RSF fibers and b) CNT/RSF fibers. a,b) Reproduced with permission.^[^ ^35^ ^]^ Copyright 2020, Wiley‐VCH. c,d) TEM images of an RSF gel. c,d) Reproduced with permission.^[^ ^73^ ^]^ Copyright 2009, Royal Society of Chemistry. e) CNT/SF hybrid mesoscopic networks characterized by AFM image. Reproduced with permission.^[^ ^3^ ^]^ Copyright 2021, Wiley‐VCH.

Direct imaging of fibers in real space is sometimes advantageous for obtaining detailed information regarding the fibers at the nano/micro scales. Among these techniques, scanning electron microscopy (SEM) and transmission electron microscopy (TEM) are the most common methods used to test the morphology and structure of *B. mori* silk fiber materials.^[^ ^73^ ^]^ Owing to the easy operation and availability of SEM, it has been used to examine the macrostructure and micro/nanostructure of RSF fibers. Using SEM, a focused electron beam scans the RSF fiber to provide a magnified image with topological and compositional information. As shown in **Figure** **7**a,b, although SEM can be used to characterize the structure of RSF and meso‐reconstructed RSF fibers, the nanofibrils in silk fibers are closely arranged, and obtaining a clear image of the network is difficult; therefore, higher‐resolution techniques (TEM and AFM) must be used to obtain such information. RSF fibers exhibit high toughness because *β*‐crystals are parallel to each other and arranged along the fiber axis in the crystal network. When preparing TEM samples, it is necessary to cut perpendicular to the fiber axis. As a result, the crystal will bend or deform more readily, which is not easily observed. Therefore, TEM has been used to determine the morphology of nanofibrils in the gel state.^[^ ^19^ ^]^ As shown in Figure 7c,d, Gong et al. observed that the spontaneously formed translucent gel comprised entangled fibrils hundreds of nanometers long and ≈5 nm wide.^[^ ^73^ ^]^ Moreover, they simulated the distribution of nanofibers in the fiber during the fiber‐forming process by stirring and demonstrated that shear flow impacted the formation of SF nanofibers. After shearing the RSF solution for a while, several white fiber flocs were produced. In addition to TEM, AFM high‐level imaging technology has been widely used to study the topography of silk materials. AFM can provide the height information for nanofibrils, which enables addition properties, such as the diameter of nanofibrils, to be measured. Recently, Ma et al. added CNTs to an RSF solution to form meso‐reconstructed RSF fibers with a mesoscopic hybrid network structure. They obtained a new type of humidity‐responsive conductive RSF fiber and successfully determined the structure of this mesoscopic network through AFM (Figure 7e).^[^ ^35^ ^]^

### 2.3 Construction Kinetics of Regenerated Silk Fibers: Nucleation Controlled SF Molecular Meso‐Refolding

**Figure 8:**
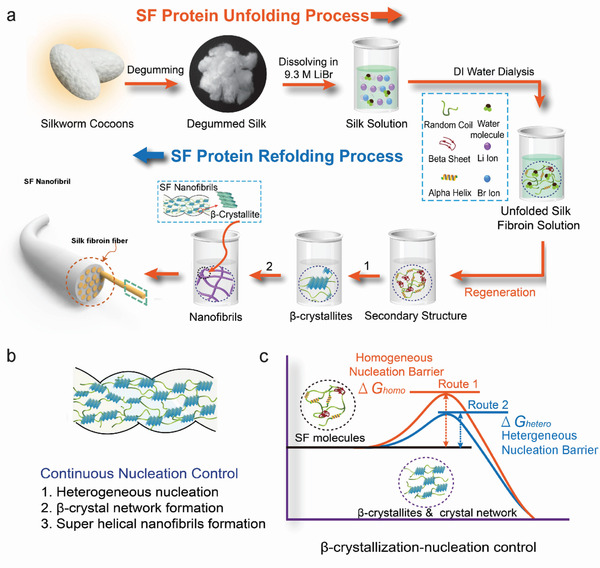
a) Illustration of the SF unfolding and refolding processes. Reproduced with permission.^[^ ^18^ ^]^ Copyright 2021, Wiley‐VCH. Degummed Silk: Reproduced with permission.^[^ ^33^ ^]^ Copyright 2014, Wiley‐VCH. Random Coil, Beta Sheet, Alpha Helix, Water Molecule, Unfolded Silk Fibroin Solution, *β*‐crystallite, SF Nanofibrils: Reproduced with permission.^[^ ^46^ ^]^ Copyright 2019, Wiley‐VCH. Silk fiber model: Reproduced with permission.^[^ ^35^ ^]^ Copyright 2020, Wiley‐VCH. b,c) Nucleation control for the formation of *β*‐crystallites & crystal network. SF molecules, *β*‐crystallites & crystal network, Continuous Nucleation Control: Reproduced with permission.^[^ ^46^ ^]^ Copyright 2019, Wiley‐VCH.

The dissolution of *B. mori* silk fibers is correlated to an unfolding process of SF molecules while the solidification of SF from solutions is associated with a refolding process of SF molecules. In the refolding process of unfolded SF molecules (**Figure** **8**a), the dominant mesoscopic hierarchical structures formed are *β*‐crystallites, crystal networks, and nanofibrils. Nucleation controls the formation of these meso‐structures.^[^ ^18^, ^54^, ^74^ ^]^ Therefore, to enhance the performance of SF fibers, the nucleation process associated with the mesoscopic hierarchical structures in the refolding process of SF molecules may be controlled. Controlling the molecular nucleation process by altering the refolding conditions, i.e., varying the shear rate, concentration of SF solutions, or evaporation rate, or inducing the intermolecular nucleation via foreign substrates/seeds or external macromolecules will alter the route of reconstruction, thus enabling the mesoscopic structures of flexible materials to be reconstructed.^[^ ^18^ ^]^ In general, any such process is termed *meso‐reconstruction* of flexible materials.

#### 2.3.1 *β*‐Crystallite Nucleation Kinetics

Crystallization is generally consisted of two key processes: nucleation and crystal growth.^[^ ^75^, ^76^, ^77^, ^78^ ^]^ Nucleation is a process in which the reduction in volume free energy and the increase in surface free energy compete. This occurs only if the nucleation barriers (ΔG*) are overcome, and crystal nuclei reach a critical size (*Rc*).^[^ ^77^ ^]^ Protein crystallization is typically more complex. Because *β*‐crystallites are very small, growth readily occurs; thus, nucleation is the rate‐dependent factor.^[^ ^31^ ^]^ According to the mesoscopic hierarchical structure of SF materials (Section 2.1),^[^ ^19^ ^]^ the formation of the mesoscopic crystalline networks follows a “continuous nucleation model” (Figure 8b),^[^ ^19^, ^54^ ^]^ In detail, it is involved with four steps. First, SF molecules fold into secondary structures (*β*‐sheets), in which hydrogen bonds are the main interactions that maintain the structure. Second, the *β*‐sheets of adjacent SF molecules stack through the template and grow into *β*‐crystallites. Third, a crystalline network (nanofibrils) forms by continuous intermolecular nucleation, which connects many *β*‐crystallites via amorphous chains. Fourth, nanofibrils grow further through continuous nucleation to form nanofibril networks (Figure 8).^[^ ^18^ ^]^

**Figure 9:**
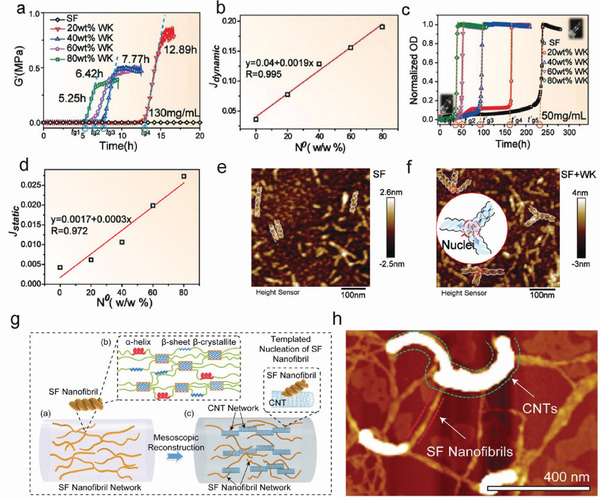
a) Rheological evolution of SF/WK hybrid solutions. b) Dynamic thermodynamic growth curve based on the rheological measurements. c) Optical density evolution of SF/WK hybrid solutions. d) Static thermodynamic growth curve based on the optical density results. e,f) AFM images of SF nanofibrils and SF/WK hybrid nanofibrils, and schematic illustration of the claw structure. a–f) Reproduced with permission.^[^ ^3^ ^]^ Copyright 2021, Wiley‐VCH. g) Schematics of SF/CNT mesoscopic network reconstruction in regenerated silk fibers. Reproduced with permission.^[^ ^35^ ^]^ Copyright 2020, Wiley‐VCH. h) AFM image of SF nanofibril networks. Reproduced with permission.^[^ ^35^ ^]^ Copyright 2020, Wiley‐VCH.

The nucleation pathway in the crystallization process not only plays a vital role in accurately controlling the size and size distribution of the crystal structure, but also determines the material alignment; various functional crystal structures can be formed by reducing the nucleation barrier of the crystal network in the material.^[^ ^19^, ^78^ ^]^ *β*‐sheets have a more compact structure than random coils. Random coils can undergo conformational transition into *β*‐sheets by a crystallization process in which *β*‐sheets are further stacked to form *β*‐crystallites. This continuous process primarily determines the formation and growth of the nanofibrils, which is also accompanied by an increase in the storage modulus (*G*′) and turbidity of the SF solution.^[^ ^54^ ^]^ Numerous studies have confirmed that nanofibril formation (*β*‐crystallite networks) is related to the dynamic induction time (*t_g_*) of *β*‐crystallite or nanofibril nucleation (**Figure** **9**a,b) and the static induction time (*t’_g_*) of nanofiber/*β*‐crystallite network nucleation (Figure 9c,d).^[^ ^19^, ^54^ ^]^ Nucleation can be classified into homogeneous and heterogeneous nucleation. The nucleation rate (*J*) can be calculated using Equations (1) and (2)^[^ ^77^, ^79^, ^80^ ^]^

(1) $$J=A\exp\left[-\Delta G^{*}f/kT\right]\;\times N^{0}$$

with

(2) $$\Delta \;G^{*}=\frac{B}{{\left(\Delta \mu \right)}^{2}}$$

where *A* and *B* are kinetic parameters, *f* is a parameter describing interaction between the templates and the nucleating phase (0<*f* ≤1; for homogeneous nucleation, *f* = 1), ∆G* is the nucleation barrier, and *∆μ* is the chemical potential difference between the parent and crystal phases. In general, *J* ≈ 1/*t_g_* ≈ 1/*t’_g_*. Based on Equation (1), *J* is proportional to the density (*N* ^0^) of the templates at the same concentration and temperature (Figure 9b,d).

In the homogeneous nucleation process, when the SF solution concentration or the equilibrium concentration of SF molecules (ionic strength, pH, temperature) changes, the crystallization process changes owing to alterations in the nucleation barrier.^[^ ^19^ ^]^ However, SF crystallization is more likely to occur via heterogenous nucleation owing to the extremely high nucleation barrier for homogeneous nucleation. In heterogeneous nucleation, foreign substances, or substrates can interact strongly with the crystalline phase to substantially reduce the protein nucleation barrier,^[^ ^77^ ^]^ leading to preferential nucleation on foreign substances or substrate surfaces and accelerated protein crystallization (Figure 8c). Foreign functional substances normally act as nucleation templates, promoting the nucleation of *β*‐crystallites and nanofibrils, which significantly reduces the gelation time (*t_g_*) compared with that of the unprocessed pure SF solution.^[^ ^46^, ^54^, ^74^ ^]^ Studying the influence of heterogeneous nucleation on the self‐assembly of SF is crucial for understanding the reconstruction of the mesoscopic structure for mesoscopic hybridization functions. Various materials, such as cellulose nanofibers (CNFs), monodisperse colloidal particles, WK, and CNTs exhibit strong template effects in the SF heterogeneous nucleation process.^[^ ^3^, ^81^, ^82^ ^]^ For example, the surface of CNFs can promote the preferential aggregation of secondary structures along the SF nanofiber axis to accelerate self‐assembly of SF along the fibril direction through hydrogen bonding. A peculiar “shish kebab” nanostructure can be formed by CNF‐directed SF assembly, resulting in excellent mechanical performance and ultrafast water transport ability while maintaining the intrinsic advantages of both CNFs and SF.^[^ ^81^ ^]^ Zhang et al. reported that WK can act as an ideal foreign substance, providing a *β*‐crystallite nucleation template that decreases the nucleation barrier and promotes SF gelation.^[^ ^3^ ^]^ Based on the AFM images of SF and SF/WK materials, the SF material exhibits a helical nanofibril structure, while the SF/WK material exhibits triangular interconnections between the SF nanofibrils (Figure 9e,f), which attributed to the WK templating.^[^ ^3^ ^]^ Notably, the interactions of carbon materials with proteins have been widely investigated because the carbon materials possess controllable polymorphism, good conductivity, and ordered surface structures.^[^ ^83^ ^]^ Ma et al. reported reconstructed SF networks with CNT templates. CNTs work as foreign substrates to promote the heterogeneous nucleation of SF *β*‐crystallite networks (Figure 9g,h).^[^ ^35^ ^]^ Shao et al. reported that the growth of SF assemblies could be guided by exfoliated graphene nanosheets, thereby promoting the formation of antiparallel *β*‐sheets along the graphene surface.^[^ ^84^ ^]^ Graphene sheets were covered with densely packed SF nanofibril layers, demonstrating that graphene can act as an effective template for the heterogeneous nucleation and crystallization of SF.

#### 2.3.2 Meso‐Reconstruction, Meso‐Doping, and Meso‐Hybridization of SF Materials

Using meso‐reconstruction/hybridization mechanism, numerous functional components (molecular and nanoseeds) can be used to fabricate novel SF hybrid fibers with new functionalities that maintain the original SF properties. Nanofunctional seeds act as heterogeneous nucleation sites to promote SF crystallization and adjust the mesoscopic hierarchical structures. The seeds are incorporated into the final SF fibers as functional components. Therefore, the hybridized SF fibers exhibit robust strength, good toughness, and additional functionalities, such as electrical conductivity and magnetic, cellular, or fluorescence properties. Notably, the functional seeds can be nanoparticles, such as QDs, upconversion nanoparticles (UCNPs),^[^ ^18^ ^]^ silver clusters (AuNCs),^[^ ^46^ ^]^ and CNTs,^[^ ^35^ ^]^ or functional macromolecules, such as wool keratin (WK),^[^ ^82^, ^96^ ^]^ bovine serum albumin,^[^ ^46^ ^]^ and polyurethane.^[^ ^37^ ^]^

**Figure 10:**
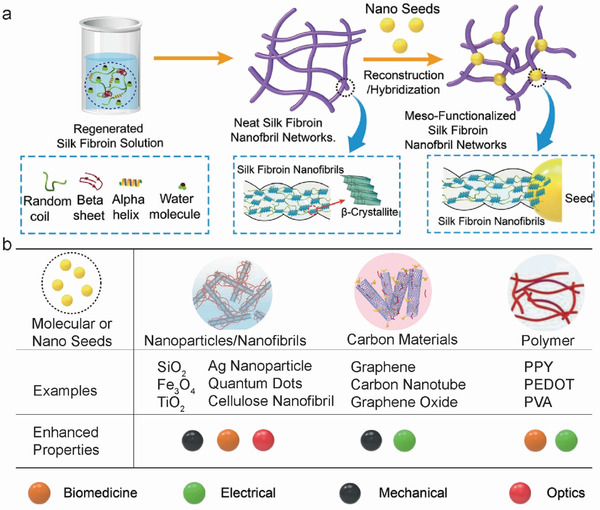
Mesoscopic reconstruction and functionalization of *B. mori* SF fibers. a) Schematic of SF mesoscopic functionalization and the impact of nanoseeds. Reproduced with permission.^[^ ^46^ ^]^ Copyright 2019, Wiley‐VCH. b) Nanoseed functionalization of SF fibers and the potentially improved properties. Molecular or Nano Seeds: Reproduced with permission.^[^ ^18^ ^]^ Copyright 2021, Wiley‐VCH. Nanoparticles/Nanofibrils: Reproduced with permission.^[^ ^85^ ^]^ Copyright 2018, Wiley‐VCH. Carbon Materials: Reproduced with permission.^[^ ^20^ ^]^ Copyright 2019, Wiley‐VCH. Polymer: Reproduced with permission.^[^ ^86^ ^]^ Copyright 2019, Wiley‐VCH. Examples: SiO_2_,^[^ ^21^ ^]^ Fe_3_O_4_,^[^ ^87^ ^]^ TiO_2_,^[^ ^88^ ^]^ Ag nanoparticle,^[^ ^89^ ^]^ quantum dots,^[^ ^90^ ^]^ cellulose nanofibril,^[^ ^91^ ^]^ graphene,^[^ ^92^ ^]^ carbon nanotube,^[^ ^20^, ^35^ ^]^ graphene oxide,^[^ ^93^ ^]^ Polypyrrole (PPy),^[^ ^94^ ^]^ Poly(3,4‐ethylenedioxythiophene) (PEDOT),^[^ ^94^ ^]^ polyvinyl alcohol (PVA).^[^ ^95^ ^]^

Regenerated silk fibers can be produced from an unfolded SF solution by the SF protein refolding process, which results in the formation of mesoscopic hierarchical structures by continuous nucleation (**Figure** **10**a).^[^ ^18^ ^]^ Among these processes, the rate‐limiting steps are *β*‐crystallite and network or nanofibril formation.^[^ ^19^, ^54^ ^]^ Therefore, functional molecular or nanoseeds endow the SF fibers with different ultimate structures, resulting in programmable fiber functionalities or performances, as shown in Figure 10.

Since the performance of meso‐materials is determined by the mesoscopic structures, the mesoscopic structures can be modified to obtain an enhanced performance. Meso‐functionalization endows natural SF materials with specialized or designed functionalization at the mesoscopic scale. More specifically, meso‐functionalization covers the following three approaches: i) meso‐reconstruction, ii) meso‐doping, and iii) meso‐hybridization. Meso‐reconstruction of the SF mesoscopic hierarchical structures is most common. It refers to any approach that causes a significant change in the hierarchical structures by rerouting the refolding process of SF. This includes any methods that can influence the nucleation process, including nucleation templating and application of a thermodynamic driving force or shearing force.^[^ ^76^, ^97^, ^98^ ^]^ Meso‐doping and meso‐hybridization are associated with meso‐nucleation templating. Meso‐doping refers to doping nanoseeds, such as QDs, metal nanoclusters, fluorescent dyes, and UCNPs into the SF mesoscopic networks.^[^ ^18^ ^]^ For example, mesoscopic doping of AuNCs into the SF hieratical networks leads to a faster switching rate and significantly enhanced performance of the silk‐based memristors, owing to the reconstructed mesoscopic electronic structures.^[^ ^46^ ^]^

As another example, Ma et al. developed a CNT/SF fiber with hybrid networks via wet‐spinning technology. In the formed mesoscopic hybrid network, the SF nanofibril network acts as a humidity‐sensitive network, whereas the CNT network acts as a conductive framework. Therefore, in addition to the individual functionalities (such as the humidity‐driven cyclic contraction of SF and conductivity of CNTs), the CNT/SF hybrid fibers exhibit new functionalities, such as electrical humidity sensing.^[^ ^35^ ^]^ Liu et al. demonstrated that CNTs provide a template for SF assembly, resulting in a higher *β*‐crystallite friction in SF/CNT fibers than in pure SF fibers. The CNT network endows the regenerated SF fibers with good conductivity and sensing ability.^[^ ^35^ ^]^

Meso‐hybridization is the rebuilding of mesoscopic networks of SF nanofibril networks by incorporating macromolecules into meso‐hierarchical networks of SF materials via hetero‐molecular nucleation templating.^[^ ^3^ ^]^ This enables partial structures of macromolecules, e.g., WK, to be incorporated into the meso‐hierarchical networks of SF materials.^[^ ^3^ ^]^ In the case of meso‐hybridization of SF by WK, molecular spring‐like *α*‐helices of WK molecules are built into the meso‐hierarchical networks of SF materials. This causes a significant enhancement in the elasticity of SF materials.^[^ ^3^ ^]^ This hybrid meso‐network not only possesses the functionalities of both individuals, but also exhibits new functionalities.

Evidently, both meso‐doping and meso‐hybridization will be coupled with meso‐reconstruction, whereas meso‐reconstruction may not involve meso‐doping or meso‐hybridization.

Based on the general heterogeneous nucleation models,^[^ ^54^, ^76^, ^99^ ^]^ nanoseeds can accelerate the formation of *β*‐crystallite networks by lowering the nucleation barrier, as shown in Figure 8c.^[^ ^18^ ^]^ For SF materials, the formation of the mesoscopic structure is facilitated by the addition of nanoseeds, which reduces the SF solution gelation time.^[^ ^54^ ^]^ The total fraction of *β*‐conformations (*β*‐sheets and *β*‐crystallites) of composite SF fibers with nanoseeds exceeds that of pure SF fibers, which confirms that nanoseeds promote *β*‐crystallite formation.^[^ ^35^ ^]^ Furthermore, AFM analysis of the mesoscopic structure also confirmed that the new nanofibril networks were reconstructed with firmly embedded nanoseeds (Figure 9e). Therefore, new functionalized silk fibers can be synthesized through the reconstruction of stable mesoscopic structures by embedding molecular or nanoseeds in SF materials. As shown in Figure 10b, certain types of nanoseeds have been successfully doped into the hierarchical mesoscopic structures of SF fibers, thus promoting many potential applications.

2D carbon materials, such as graphene, can also promote the mechanical performance of SF hybrid fibers, yielding a breaking stress of 697 ± 22 MPa, which is a 58.7% improvement in the breaking stress of pure regenerated silk fibers.^[^ ^92^ ^]^ SF/carbon hybrid materials have promising applications in tissue engineering and wearable electronics such as conductive wires, pressure sensors, and humidity sensors, as well as in tissue engineering. Similar enhanced performance can be observed in SF hybrid fibers containing conductive conjugated polymers, such as PPy and poly(3,4‐ethylene‐dioxythiophene) (PEDOT). These functionalized SF fibers exhibit better electrical and thermal properties,^[^ ^94^ ^]^ thereby facilitating potential applications in conductive textiles and biological engineering. Moreover, nanoparticles and nanofibrils can be introduced to the unfolded SF solutions to increase the mechanical performance of the final silk fibers. The addition of SiO_2_ nanoparticles at a concentration of 0.1 w/w% can provide heterogeneous nucleation sites to initiate the formation of *β*‐crystallite, thus enhancing the breaking stress by ≈50%.^[^ ^21^ ^]^ Moreover, by embedding SF with magnetic nanoparticle (such as Fe_3_O_4_), novel magnetization functionality can be added to SF fibers.^[^ ^87^ ^]^ Similarly, the optical performance can be enhanced by the addition of CdS QDs.^[^ ^100^ ^]^ The reassembled fibers are mechanically stable, biodegradable, and weavable, which are beneficial for various applications, including tissue engineering and biocompatible devices.

## 3 Meso‐Functionalization of SF Fibers by Artificial Spinning

Compared to directly modified natural *B. mori* silk fibers, functional SF‐based fibers can be prepared by artificial spinning methods using a silk‐based spinning solution. A suitable spinning strategy is the most important factor for fiber preparation. Consequently, the spinning method for meso‐reconstructed functional SF‐based fibers must be carefully selected considering various factors, such as the properties of additional materials, hybrid material performance, spinning stability, fiber structure, and final functionality of the fiber. This section presents four representative artificial spinning fabrication techniques: wet spinning, dry spinning, electrospinning, and microfluidic spinning.

### 3.1 Wet Spinning

**Figure 11:**
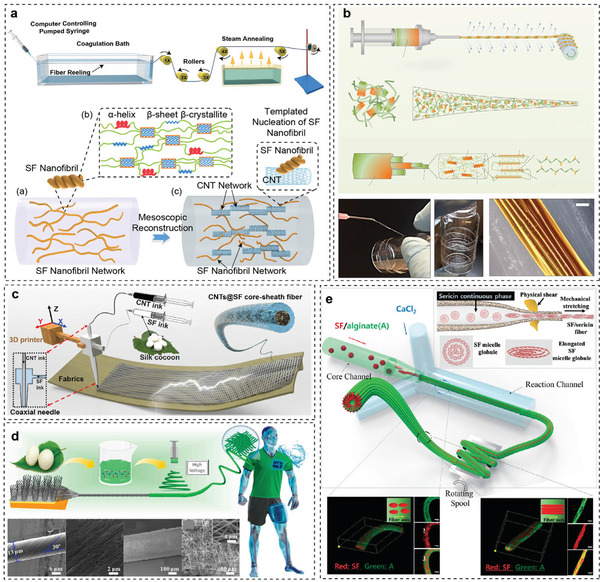
Artificial RSF spinning method. a) Wet spinning applied to obtain a hybrid mesoscopic reconstructed RSF using CNTs as nucleation nano seeds. Reproduced with permission.^[^ ^35^ ^]^ Copyright 2020, Wiley‐VCH. b) Dry spinning applied to fabricate an RSF fiber with good mechanical performance. Reproduced under the terms of the CC‐BY license.^[^ ^105^ ^]^ Copyright 2017, the Authors. Published by Springer Nature. c) 3D printing applied to obtain a core–sheath‐structured functional RSF fibers. Reproduced with permission.^[^ ^41^ ^]^ Copyright 2019, Cell press. d) RSF nanofibers obtained by electrospinning. Reproduced with permission.^[^ ^106^ ^]^ Copyright 2018, American Chemical Society. e) Meso‐hybridized RSF/alginate fibers prepared by microfluidic spinning Reproduced with permission.^[^ ^107^ ^]^ Copyright 2016, the Royal Society of Chemistry.

The most common spinning technique for silk‐based fiber spinning is the wet‐spinning process, which is shown in **Figure** **11**. Compared to synthetic fibers, natural *B. mori* silk fibers are typically spun under milder conditions, i.e., in silk glands. Several factors should be considered for wet spinning of functional RSF fibers, including the silk solution, coagulation bath, and post‐treatment.

#### 3.1.1 SF Solutions

Natural silk comprises SF and sericin. SF endows the silk with excellent mechanical properties, whereas sericin is a binder. Therefore, most scientific research is based on RSF. Based on the composition of silk, the silk must first be degummed to obtain the RSF. Because sericin exhibits good water solubility, silk can be degummed using high‐temperature aqueous or alkaline solutions (NaHCO_3_, Na_2_CO_3_) to obtain SF. To obtain an SF solution with appropriate rheological properties, a suitable solvent must be selected to dissolve the silk protein. The dissolution systems for silk proteins are mainly divided into three categories: organic solvents, ionic liquids, and aqueous solvents. SF solutions with different degrees of solubility can be obtained by dissolving SF through different dissolution systems, which is a prerequisite for the realization of meso‐reconstructed RSF fibers. Most organic solvent dissolution systems are not ideal due to their expense and toxicity. Further, they may cause severe degradation of SF and poor mechanical properties. Although some studies have revealed that fibers prepared by organic solvent dissolution systems have excellent mechanical properties, the fiber diameter is too large;^[^ ^101^ ^]^ therefore, organic solvents are not suitable for large‐scale industrial production. In ionic liquid dissolution systems, the ionic liquid can destroy the hydrogen bonds between the silk protein molecular chains, thereby destroying the structure of the silk protein molecule and dissolving it. Furthermore, the high temperature of the ionic liquids may cause pyrolysis of the silk protein molecular chain, which is not conducive to the preparation of regenerated silk fibers with excellent mechanical properties. Although some ionic liquids that dissolve silk at room temperature have recently been identified, the requirements for dissolution are relatively high.^[^ ^102^ ^]^

Aqueous solvent dissolution systems are more environmentally friendly than the two aforementioned dissolution systems. They can obtain a protein chain solution with a high packing fraction, thereby ensuring the production of RSF fibers with excellent toughness.^[^ ^19^ ^]^ In the spinning process, SF must possess suitable rheological properties. In natural silk glands, the concentration of the viscous solution can reach up to 30%. When an RSF aqueous solution is used as the original solution, its concentration is considerably lower. Therefore, in addition to dissolution and dialysis, reverse dialysis is required to prepare RSF aqueous solutions to increase the concentration of the regenerated silk solution. The silk solution concentration after reverse dialysis can be controlled by the dialysis time. For example, when the concentration of the silk solution was <13%, fibers could not be formed; however, when the concentration of the silk solution was ≈19%, fibers could be formed easily.^[^ ^103^ ^]^ By extending the reverse dialysis time of silk, the concentration of the silk solution could reach >39.8%, even up to 55%.^[^ ^19^ ^]^ When the solution concentration is sufficiently high, the silk solution readily forms a gel, and the formed fiber exhibits poor mechanical properties and breaks easily. However, the advantage of a high‐concentration silk solution is that it can be diluted and mixed with other functional materials to prepare functional regenerated silk fibers. To endow SF materials with atypical properties (e.g., electrical conductivity), Liu et al. used a high‐concentration silk solution and conductive materials to meso‐reconstruct the SF material.^[^ ^35^ ^]^ As shown in Figure 11a, the basic structural entities of SF nanofibrils are *β*‐crystallite networks, and SF nanofibril networks are one of the paramount meso‐structures within the material. To acquire functional RSF fibers, SF nanofibril network structure was altered by CNTs, which were adopted as functional nanoseeds. And an environmentally friendly wet‐spinning method was used to fabricate conductive meso‐reconstructed RSF hybrid fibers.

#### 3.1.2 Coagulation Bath

The coagulation bath is an important factor that affects fiber formation, and the presence or absence of a coagulation bath is the primary difference between wet and dry spinning. Several aqueous solvents, organic solvents, and ionic liquids can be used as coagulation baths, such as ethanol, methanol, isopropanol, *n*‐butanol, glycerin, glycol, and various sodium, zinc, potassium, magnesium, and ammonium salts.^[^ ^104^ ^]^ The coagulation rate of the fiber in these coagulation baths directly affects the structure of the RSF fiber, thereby affecting the final performance of the fiber. If the coagulation rate is insufficient, it is difficult to shape the fiber. However, if the solidification rate is excessive, the virgin RSF fibers become brittle. In addition to the type of solvent in the coagulation bath, factors such as solution concentration and temperature also affect the morphology and mechanical properties of the fibers.

#### 3.1.3 Post‐Treatment

After RSF fibers are coagulated in the coagulation bath, the fibers are relatively fragile owing to the low crystallinity and poor orientation function of crystallites in the crystal network.^[^ ^19^ ^]^ Therefore, poststretching in an air gap or in a solution (post‐treatment) is required. The fibers are stretched along the fiber axis in a gas atmosphere gap or solution. Under external force, the crystallinity of the fiber and orientation function of the crystal network are significantly enhanced. Based on the literature, postdrawing treatment can significantly improve the morphology and mechanical properties of the fibers. Regarding the fiber morphology, the surface of post‐treated RSF fibers exhibits a smooth and lustrous appearance. Regarding the mechanical properties of the fiber, Zhou et al. implemented different postdraw ratios on as‐spun fibers; the results revealed that increasing the stretch ratio gradually increased the molecular arrangement, improved the orientation of the *β*‐sheet secondary structure, and increased the level of crystallinity.^[^ ^104^ ^]^ The above‐mentioned changes in the mesoscopic structure should directly cause an improvement in the breaking stress of the fibers.

### 3.2 Dry Spinning

Dry spinning is also a vital technique for the mesoscopic engineering of RSF fibers. The dry spinning of functional RSF fibers is very similar to the natural *B. mori* silk fibers spinning process. Dry spinning is similar to wet spinning in that it requires fiber pretreatment, spinning solution preparation, and post‐treatment. However, the main difference from wet spinning is that the fiber is formed in air instead of a coagulation bath.

For dry spinning, the solvent for the spinning solution should also have a strong ability to destroy hydrogen bonds and physical crosslinks but have a minimal effect on peptide bonds to avoid excessive degradation of protein molecular chains. Artificial RSF fibers prepared by dry spinning use various dissolution systems,^[^ ^105^ ^]^ such as 1,1,1,3,3,3‐hexafluoro‐2‐propanol (HFIP), hexafluoroacetone (HFA), *N*‐methyl morpholine N‐oxide (NMMO), lithium bromide (LiBr)/H_2_O, and calcium chloride (CaCl_2_)/formic acid,^[^ ^108^ ^]^ to dissolve and destroy the fibers and the multilayer structure. Functional RSF fibers are prepared by blending or mixing with functional materials.

Importantly, Kaplan et al. used an HFIP dissolution system to partially dissolve silk fibers and prepared microfibrils (diameter: 5–50 µm and contour length: 50–500).^[^ ^109^ ^]^ As shown in Figure 11b, the spinning dope consisting of microfibrils and HFIP had an appropriate viscosity, concentration, consistency, and stability, making the regenerated silk solution easier to spin. As the spinning dope was extruded from the spinneret, the HFIP was volatilized in the air, and the external force applied during drawing resulted in highly oriented uniform fibers with good mechanical properties. The fibers had excellent performance because the RSF retained the structural hierarchy and well‐organized silk nanofiber structure of natural silk.^[^ ^105^ ^]^ This research provides a new concept for the engineering regulation of silk on a mesoscopic scale. This semidissolving system can be combined with an ordinary dissolving system to prepare functional RSF fibers with excellent mechanical properties.

### 3.3 3D Printing Spinning

With the development of printing equipment and nanomaterials, 3D printing has rapidly developed into an important and advanced manufacturing technology for biomedical field and electronics field.^[^ ^110^ ^]^ As early as 2008, Kaplan et al. used SF ink to deposit layer‐by‐layer silk fiber array with a diameter of 5 mm, that is, a 3D printed silk fiber‐based scaffold.^[^ ^111^ ^]^ The 3D printing technology has advantages in complex geometric figures and precise spatial scaffold structures design, thereby plays an important role in biomedical engineering field by combing with the good biocompatibility with SF materials.^[^ ^112^ ^]^ Recently, the 3D printing technology has also proven to be an effective method to fabricate fiber‐based functional devices. The multicomponent or skin‐core structure fiber can be obtained by replacing the printing inkjet head, thereby realizing the function of a fiber‐based functional device.^[^ ^113^ ^]^ Zhang et al. used a 3D printing method (Figure 11c) to prepare composite fibers with SF as the shell and CNTs as the core.^[^ ^41^ ^]^ The composite fibers exhibited conduction, sensing, energy collection, and good biocompatibility.

### 3.4 Electrospinning

Based on the good biocompatibility and biodegradability of *B. mori* silk fibers, silk nanofiber bundles, and membranes prepared by electrospinning have received extensive research attention in biomedicine, smart wearables, and other fields. Unlike wet and dry spinning, electrospinning employs a high‐voltage electric field to draw the RSF spinning drop. The obtained fibers are mostly nanometer or submicrometer fibers with relatively poor mechanical properties. For both electrospinning and dry spinning, the regenerated silk protein spinning solution obtained by dissolving the silk is drawn in air, and the silk fibers are formed after the solvent is volatilized. Numerous research groups have conducted comprehensive research on the process conditions and parameters of electrospinning, such as the concentration of the spinning solution, conductivity, electric field strength, injection rate, and receiving distance. In recent years, numerous silk nanofibers with good mechanical properties and functionality have been prepared by adding functional materials (e.g., CNTs, graphene, silver nanoparticles, gold nanoparticles, and other particles) to the RSF solution.^[^ ^19^, ^35^ ^]^ In addition to the above‐mentioned electrospinning research, Zhang et al. prepared lightweight CNT@silk wires on a substrate of CNT fiber bundles (yarn) using silk nanofiber bundles prepared by electrospinning as the shell layer (insulating material).^[^ ^41^ ^]^ The wires exhibited good resistance to humidity, and the coating made the wires splash‐resistant, enabling their use in smart clothes (Figure 11d).^[^ ^106^ ^]^

### 3.5 Microfluidic Spinning

Unlike traditional spinning methods, microfluidic spinning can be conducted under normal temperature and pressure conditions and can be used to control the fibrous shape and size. This spinning method utilizes the microscale flow of liquid in microchannels that can be precisely controlled. Because it is difficult to control the fibers microscopically using existing spinning methods, microfluidic spinning exhibits good potential applicability in the development of new functional fibers. Natural silk has a highly ordered hierarchical structure, which provides significant mechanical strength. A bionic microfluidic chip, which simulates the geometric shape of silk glands to form an improved hierarchical directional structure, is used to reconstruct the regenerated silk fiber material by microfluidic spinning.^[^ ^107^ ^]^

By adjusting the concentration of silk in a suitable solvent system, a suitable RSF spinning drop can be obtained, and the microfluidic spinning method can be used to produce new RSF fibers. Some studies have extensively analyzed the influence of the structural parameters of the microfluidic chip, the liquid flow rate in the chip, the properties of the silk solution, and other factors on the structural, mechanical, and biological properties of the regenerated RSF fibers. Owing to the excellent properties of *B. mori* silk fiber, it can also be added to other materials to improve the performance of composite fibers (Figure 11e).^[^ ^107^ ^]^

## 4 Meso‐Functionalized Silk Fibrous Devices and Applications

By combining the intrinsic merits of SF with functionalized properties during regenerative spinning, regenerated silk fibers can be used as smart fiber devices, such as sensors, actuators, optical fibers, and energy harvesters. These silk fiber devices can be applied in personal healthcare, soft robotics, light guidance, and mobile energy harvesting. In this section, various silk fiber devices are discussed, and the effect of meso‐functionalization of the SF structure on the performance of silk fiber devices is summarized.

### 4.1 Fiber Sensors and Actuators

#### 4.1.1 Pressure and Strain Sensors

SF pressure and strain sensors are mesoscopic electronic devices that can convert external mechanical signals into easily readable electrical values. Through meso‐reconstruction, the mechanical and electrical performance of SF can be manipulated, which results in SF pressure/strain sensors with controllable sensing performance.^[^ ^20^, ^24^, ^114^, ^115^ ^]^ Here, two types of sensors based on different detection principles, i.e., resistance‐ and capacitance‐type sensors, are discussed, and the sensing mechanism and recent developments in silk pressure/strain sensors are summarized.

Resistance‐type pressure/strain sensors convert mechanical force into a change in the resistance signal, normally resulting from the contact resistance variation during stretching and recovery. Owing to the robust mechanical and electrical insulation properties, meso‐functionalization has been applied to engineer SF materials with programmable mechanical strength and electrical conductivity. Mesoscopic doping of conductive substances or molecules, such as CNTs or WK, into meso‐reconstructed SF materials has been shown to enhance the mechanical properties of prepared fibers^[^ ^35^ ^]^ and films^[^ ^3^ ^]^ as well as introduce new electrical properties.

Liu et al. manipulated the *β*‐crystallite network structure by rebuilding the *α*‐helices of WK molecules in the hierarchical mesoscopic structure of SF molecules, which altered the mechanical properties (such as the Young's modulus) because of the spring structure of *α*‐helices. Moreover, CNTs can serve as nanotemplates to reconstruct hierarchical mesoscopic structures and adjust the material resistance via mesoscopic hybridization. A prepared SF hybrid conductive material was further fabricated into a resistance‐type pressure sensor by combining a surface‐patterned film with another conductive layer. It exhibited high sensitivity to applied pressure (41.3 kPa^−1^ under 250 kPa). This type of SF–WK meso‐hybrid sensor can be utilized to monitor a variety of motion and physiological signals, such as vocal cord vibrations, pulse fluctuations, and joint movements and to promote sophisticated human–machine interactions for personalized healthcare, such as blood vessel elasticity evaluation and dynamic blood pressure prediction based on the pulse wave signals.^[^ ^3^ ^]^

Carbonization is another technology that can convert insulating silk materials into electrically conductive and pressure/strain sensitive materials.^[^ ^116^, ^117^ ^]^ During carbonization, silk fabrics transformed *N* elements into *N* substituents in graphite or amorphous layers,^[^ ^118^ ^]^ showing good electrical conductivity (≈140 Ω sq^−1^). After encapsulation, the carbonized conductive silk fabric showed a high sensitivity to applied strain, i.e., gauge factors (GFs) of 9.6 and 37.5 were obtained at stretching strains of 0–250% and 250–500%, respectively.^[^ ^116^, ^117^ ^]^

**Figure 12:**
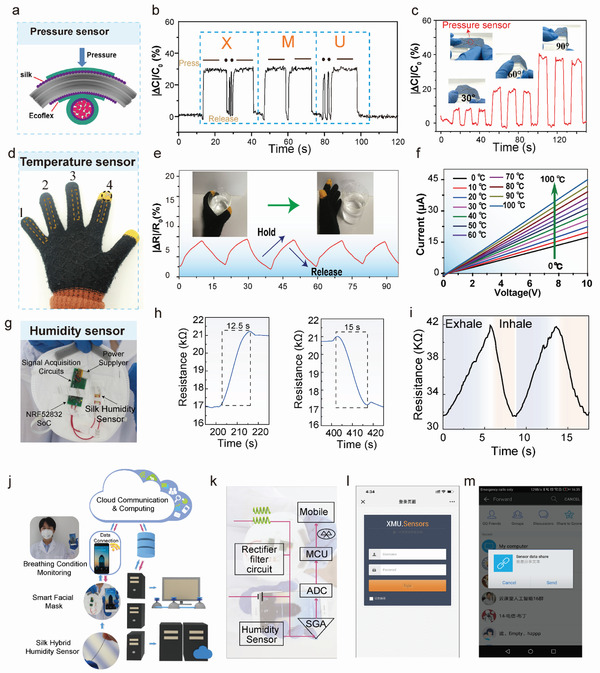
*B. mori* silk fiber sensors. a) Illustration of a silk fiber pressure sensor. b) Signal from the silk pressure sensor when tapping “X,” “M,” and “U” in Morse code. c) Bending monitoring when the pressure sensor was bent at different angles. d) Image of a smart knitted glove embedded with a silk fiber temperature sensor array. e) Electrical signals when the smart glove touched a beaker filled with hot water. f) Current–voltage signals when the silk temperature sensor was placed in different temperature environments. a–f) Reproduced with permission.^[^ ^20^ ^]^ Copyright 2019, Wiley‐VCH. g) Smart facial mask with embedded silk humidity sensor. h) Response and recovery times of the silk humidity sensor. i) Resistance signal when the smart mask wearer exhaled and inhaled. j) Schematic of the cloud platform for human respiratory monitoring. k) Flow chart of electrical signal processing. l,m) Real‐time mobile monitoring platform. g–m) Reproduced with permission.^[^ ^35^ ^]^ Copyright 2020, Wiley‐VCH.

Capacitance‐type pressure sensors have been widely developed owing to their controllable structure and stable performance.^[^ ^119^, ^120^, ^121^ ^]^ The capacitance signal response (*C* ≈ *εS*/4*πkd*) normally originates from variations in the dielectric layer (*d*) and contact area (*S*). For a textile capacitance‐type sensor, each interweaving point of the well‐designed weft and warp yarn can function as a sensing unit,^[^ ^10^ ^]^ as shown in **Figure** **12**a. Inspired by this, a silk fiber capacitance‐type sensor was fabricated using two conductive silk composite yarns acting as electrodes, where the Ecoflex coating layer on the yarn surface acted as the dielectric layer, exhibiting a sensitivity of 0.136 kPa^−1^.^[^ ^20^ ^]^

Because of the good sensitivity of wearable silk pressure/strain sensors, tiny movements from human motion and health information can be detected when sensors are embedded in intelligent gloves or clothes. For example, a silk capacitance‐type sensor integrated in an intelligent glove was applied to monitor tapping or touching signals for Morse code detection.^[^ ^20^ ^]^ The Morse code of specific characters (such as “X,” “M,” and “U”) typed by the smart glove can be recorded by the generated electrical signals, providing readable information after decryption by a translator (Figure 12b). In addition, silk fiber strain/pressure sensors can be used to detect human wrist pulses, speaking, joint bending, and muscle stretching. In addition to pressure/strain signals, other mechanical stimuli, such as bending motions, can be monitored by silk sensors (Figure 12c). In summary, silk fiber pressure/strain sensors exhibit excellent weavability, flexibility, sensitivity, and biocompatibility, showing promise for potential applications in smart textiles.

#### 4.1.2 Temperature Sensors

Natural *B. mori* silk fibers can be functionalized with temperature sensing properties by embedding functional nanomaterials into the fibers or transforming silk into carbon materials at high temperatures. Zhang et al. reported a temperature sensor containing silk‐nanofiber‐derived carbon fiber membranes. This silk fiber‐based sensors can be mass‐assembled for temperature distribution monitoring or combined with pressure sensor arrays for dual functionalities.^[^ ^122^ ^]^ Liu et al. embedded functional CNTs and ionic liquids in silk fiber gaps, then encapsulated the fibers with durable polymers to achieve a fiber‐based temperature sensor.^[^ ^20^ ^]^ The silk fiber sensor can be easily sewn into a commercialized knitted glove, as shown in Figure 12d. This smart glove can be applied to detect the temperature of the contacting object, such as a beaker containing hot water (Figure 12e). The fibrous silk temperature sensor showed a linear response with changing voltage (Figure 12f), thus facilitating its wide potential applications in smart textiles.

#### 4.1.3 Humidity Sensors

SF contains many hydrophilic domains (amorphous areas) and hydrophobic domains (crystallized areas) and shows an inner stress response under changes in humidity.^[^ ^123^ ^]^ This humidity responsiveness makes SF an excellent material for humidity sensors after meso‐functionalization. A regenerated silk fiber humidity sensor was developed by constructing an SF/CNT hybrid mesoscopic network using wet‐spinning technology.^[^ ^35^ ^]^ The SF/CNT hybrid fibers showed an electrical response when the humidity changed because of the electrical channel of the CNT networks and the humidity‐induced cyclic contraction of SF. The regenerated fibers provided a good humidity response of 58.73 RH and a short response and recovery time when the environmental humidity changed, as shown in Figure 12h. Therefore, the sensors can be used for human respiration monitoring when embedded in a human facial mask, as shown in Figure 12g. When the wearer inhales and exhales, the humidity inside the mask decrease and increase, generating a decreasing and increasing resistance signal, respectively (Figure 12i). By combining the sensor with a flexible electrical board for data processing and wireless transmission, the data collected from the smart facial mask can be uploaded to a cloud platform, as shown in Figure 12j. This system can also be connected to a mobile phone to display real‐time human respiration information (Figure 12k–m). This wearable silk sensor‐based platform can help accelerate remote medical monitoring and diagnosis, provide resources for big data analysis in disease research, and promote personalized wearable medical healthcare.^[^ ^35^, ^124^ ^]^

#### 4.1.4 Silk Fiber Actuators

SF is considered a potential material for actuators because of its good mechanical properties and adjustable moisture absorption properties. As mentioned in Section 4.1.3, SF contains many hydrophilic domains and exhibits an inner stress response under changes in humidity. The humidity‐induced actuation of spider dragline silk is larger than that of state‐of‐the‐art CNT microactuators (250° mm^−1^).

This excellent actuation property enables the spider web to maintain its geometric configuration in a changing environment and even allows the spider to perceive the external load imposed on the web.^[^ ^25^ ^]^ *B. mori* silk fibers have a molecular structure similar to that of spider silk proteins^[^ ^125^ ^]^ and the ability to lift a small load through humidity‐induced cyclic contraction and expansion. Therefore, many studies have been conducted on humidity‐triggered silk fiber (yarn) actuators. Viney et al. reported that the maximum supercontraction stress of a spider dragline (*Nephila clavipes*) reached 22% of the breaking strength when it was initially exposed to humidity.^[^ ^126^ ^]^ Agnarsson et al. further researched the supercontraction of dragline silk and revealed that dragline silk exhibits two different responses to moisture.^[^ ^127^ ^]^

**Figure 13:**
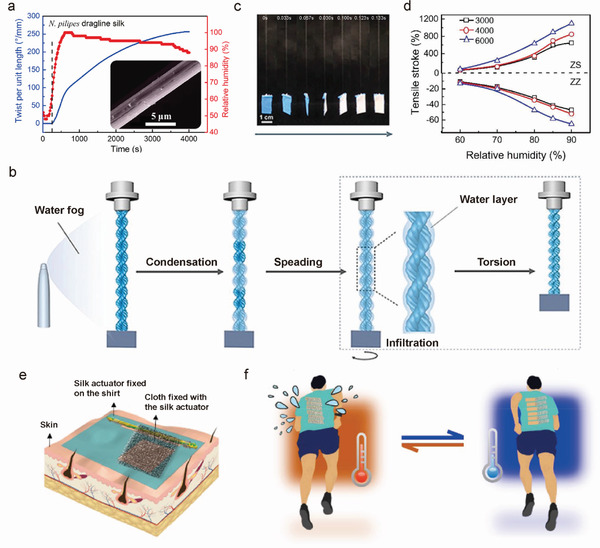
Silk fiber actuators. a) Humidity‐induced torsional actuation of spider dragline silks. Reproduced with permission.^[^ ^130^ ^]^ Copyright 2019, the Authors. Published by American Association for the Advancement of Science. b) Schematic of a water‐fog‐activated silk actuator. c) Images of the working process of the silk actuator (*p* is the screw pitch). b,c) Reproduced under the terms of the CC‐BY license.^[^ ^42^ ^]^ Copyright 2020, the Authors. Published by Wiley‐VCH. d) Dependence of tensile stroke on relative humidity for silk muscles with different twist densities. Reproduced with permission.^[^ ^132^ ^]^ Copyright 2019, Wiley‐VCH. e) Structure of a silk actuator sewn onto a T‐shirt. f) Demonstration of the application of human‐sweat‐driven silk yarn for air conditioning textiles. e,f) Reproduced with permission.^[^ ^133^ ^]^ Copyright 2019, Springer Nature.

When water initially penetrates silk, it mainly interacts with hydrophilic amino acids in the random coils. The rupture of these relatively weak hydrogen bonds causes the filaments to relax slightly, but the glycine‐rich linker regions maintain the overall orientation; consequently, the random coil network maintains its orientation. When the humidity exceeds the critical threshold of ≈70%, water penetrates the glycine‐rich linker regions and breaks their hydrogen bonds. This is sufficient to reconfigure the SF in a higher entropy direction and cause the entire silk to suddenly shrink lengthwise, while the overall volume expands. Based on this analysis, Ling et al. established a quantitative molecular mechanical model to describe the interaction between *Antheraea pernyi* silks and water molecules, both experimentally and theoretically.^[^ ^128^ ^]^ Owing to its remarkable performance in humid environments, many researchers have used it to develop humidity‐stimulated artificial muscles or actuators. Blackledge et al. studied the powerful cyclic contractions of *N. clavipes* under humid conditions and found that the contractions under repeated humidity changes can generate 50 times more power than the equivalent mass of human muscle.^[^ ^129^ ^]^ More interestingly, Buehler et al. reported that spider dragline silk shows a torsional deformation of >300° mm^−1^ in humid environments. When the relative humidity reached a threshold of ≈70%, the dragline silk began to produce large distortions, as shown in **Figure** **13**a. Simulations of dragline silk proteins showed that the novel torsional performance results from the proline in MaSp2. The large proline rings also contribute to steric repulsion and the destruction of hydrogen bonds in the molecule.^[^ ^130^ ^]^ Li et al. reported that twisted spider dragline silk yarns can contribute to a substantially enhanced lengthwise contraction of 60% and an isometric stress of 11 MPa after wetting.^[^ ^131^ ^]^ The phenomena of directional twisting and supercontraction were only observed in spider silk, not in silkworm silk. However, researchers have taken advantage of the cyclic response of *B. mori* under humidity and the yarn structure to design yarn actuators. As shown in Figure 13b, using a twisted multistrand yarn structure, a self‐balanced torsional silk muscle can twist under changes in humidity and act as a torsional silk muscle. To achieve a self‐balanced yarn structure, the twist direction of a single fiber should be S, and the twist direction of the two parts of the fiber should be Z. Further, the silk yarn can twist in humid environments because fiber volume expansion causes individual fiber segments to untwist, resulting in a torque that causes an increase in the twisting of the two fibers in the Z direction because the twisting direction of the individual fibers is opposite to that of the two fibers. Ling et al. employed twisted force‐reeled silks and twisted silk fibers into double‐helical actuators with a programmable actuation power of 0.77–2.1 W kg^−1^,^[^ ^42^ ^]^ as shown in Figure 13c. Liu et al. plied twisted silk fibers into a yarn and demonstrated a reversible torsional stroke of 547° mm^−1^ (Figure 13d). Zhang et al. fabricated an electrospun silk yarn with highly oriented nanofibers that can absorb sweat and automatically rotate under humid conditions, which enables it to be applied in air‐conditioning smart textiles, as shown in Figure 13e,f.

### 4.2 Silk Fibers in Optical Applications

Natural silk fibers exhibit excellent optical transparencies (≈90%) and reflective properties, providing silk fabrics with exceptional lustrous performance. Mesoscopic functionalized silk fibers have attracted considerable attention because of their programmable properties and superior performance in optical applications, such as fluorescent or optical fibers.

#### 4.2.1 Fluorescent Silk Fibers

**Figure 14:**
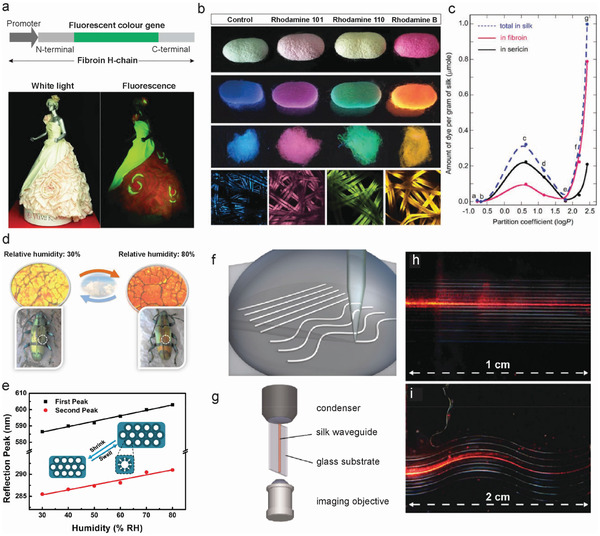
Optical applications of *B. mori* silk fibers. a) Vector structure encoded by the fluorescent color gene (green), which is flanked by the N‐terminal and C‐terminal regions of the fibroin H chain, driven by the fibroin H chain promoter. Reproduced with permission.^[^ ^134^ ^]^ Copyright 2013, Wiley‐VCH. b) Images of the color fluorescent cocoons and fibers produced by normal silkworms (as a control) and silkworms fed with various fluorescent dyes (including Rhodamine 101, Rhodamine 110, and Rhodamine B). The images in the first row were taken under white light, while the other three rows were taken under UV irradiation. c) Variation in the quantities of various fluorescent dyes in sericin, fibroin, and silk according to the partition coefficient (log P). b,c) Reproduced with permission.^[^ ^43^ ^]^ Copyright 2011, Wiley‐VCH. d) Structural color of biomimetic silk fibroin inverse opal in response to humidity. e) Relationship between the reflection peaks and environmental humidity. Reproduced with permission.^[^ ^123^ ^]^ Copyright 2013, Wiley‐VCH. f) Schematic illustration of direct‐write assembly of silk waveguides. g) Schematic diagram of a device for imaging and analyzing the lateral surface of a silk waveguide. h,i) Optical images of silk waveguides with straight and wavy structure guiding light from a He:Ne laser source. f–i) Reproduced with permission.^[^ ^44^ ^]^ Copyright 2009, Wiley‐VCH.

Because silk contains amino acids such as tryptophan, it shows weak fluorescence without any dye. To increase the fluorescence of *B. mori* silk fibers, methods such as genetic modification, feeding, and meso‐doping with luminescent materials have been developed. Compared with *B. mori* silk fibers with only weak fluorescence, fluorescent proteins, including red fluorescent protein (RFP), green fluorescent protein (GFP), and yellow fluorescent protein (YFP), emit a high quantum yield over a wide spectral range covering visible light.^[^ ^27^ ^]^ Therefore, to increase the fluorescence of silk fibers, genetic modification methods that express the fluorescent color protein in silkworms have been developed (**Figure** **14**a).^[^ ^134^ ^]^ Using vectors derived from the SF H chain gene and classical breeding methods, transgenic silkworms can produce large quantities of green, red, and orange fluorescent silk fibers. These fibers were mass‐produced and woven into fabrics by rearing over 20 000 silkworms. Another way to manipulate the fluorescence of silk fibers is to feed domesticated silkworms fluorescent dyes, resulting in the direct production of silk fibers with intrinsic color.^[^ ^134^ ^]^ Rhodamine dyes, such as rhodamine B, rhodamine 110, and rhodamine 101, were successfully absorbed in vivo by silkworms to produce cocoons with various visible and fluorescent colors,^[^ ^43^ ^]^ as shown in Figure 14b. The measured quantities of fluorescent dyes absorbed by silkworms and each of the components as a function of the partition coefficient (log P) are shown in Figure 14c. The amount of dye in the silk showed a strong relationship with log P, and more rhodamine 110 was absorbed into sericin than SF. This method of bio‐incorporating dyes into SF is a more environmentally friendly method of producing colored silk because it eliminates the need for external dyeing processes and related resources (water, energy, and other chemicals) and postproduction. SF from silkworm fibers can be produced for inverse opals with bistructural colors in the UV and visible (UV/vis), UV and infrared (UV/IR), and visible and IR (visible/IR) regions. The structural parameters can be adjusted to control the reflection peak wavelengths of the bistructural color.^[^ ^123^ ^]^ In addition, cyclic contraction of silk fibroin induced by humidity enables the structural color change, which was used for the biomimicry of longhorn beetles, as shown in Figure 14d. There is a linear relationship between the reflection peaks of the silk‐fibroin inverse opal and the relative humidity level, as shown in Figure 14e.

#### 4.2.2 Silk Optical Fibers

Silk optical fibers provide a biocompatible property that is not observed in traditional glass‐based fibers. The efficient behavior of pristine dragline silk optical fiber from *Nephila clavipes* silk was first demonstrated, then the attenuation coefficient of the straight fiber was estimated to be ≈10.5 dB cm^−1^ by manipulating them in air.^[^ ^26^ ^]^ Further, SF derived from silkworm silk is also used to manufacture mercerized waveguides with straight and wavy structures through direct ink writing technology (Figure 14f,g). The printed silk waveguide provides a high‐quality light guide and the measured losses of the straight and curved waveguide are 0.25 and 0.81 dB cm^−1^, respectively.^[^ ^44^ ^]^ The silk optical fiber images under the 633 nm He:Ne laser light (Figure 14h,i) shows that the light was coupled into the specific illuminated silk fiber. Silk optical fibers are promising candidates for biophotonic applications, which require light propagation or sensing in biocompatible media.

### 4.3 Fibrous Energy Devices

*B. mori* silk fiber is a widely used fiber resource with dielectric property. Therefore, research on silk fibers in energy harvesting is important for the development of sustainable, renewable, and wearable energy sources. Silk fibroin exhibits a piezoelectric or triboelectric effect, which enables it to be utilized for harvesting energy. Silk fiber from spider silk reveals a vertical piezoelectric coefficient of ≈0.36 pm V^−1^.^[^ ^135^ ^]^ According to triboelectric series, silk fibers have a higher electropositive performance than many artificial polymer fibers, such as polyester (PET), polyimide (PI), polytetrafluoroethylene (PTFE) fibers, and thus have been widely developed in triboelectric nanogenerators (TENGs) as well. For silk fiber energy harvester, core–sheath and sandwiched structures are mostly developed.

**Figure 15:**
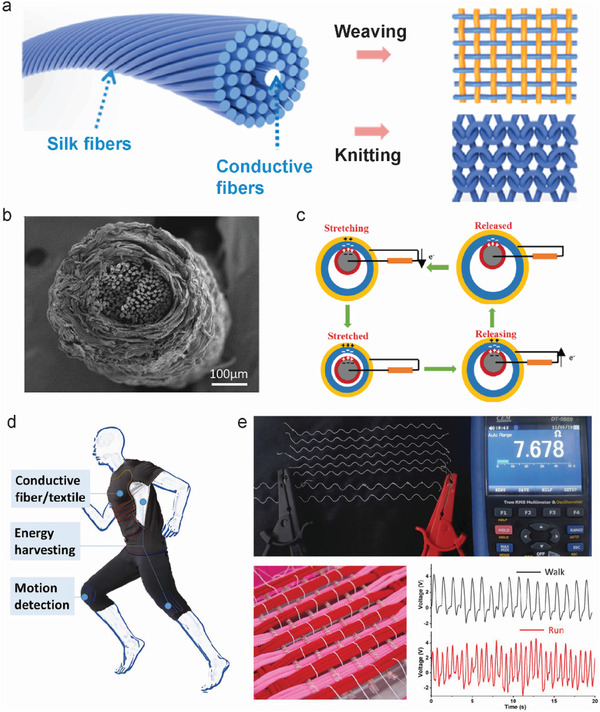
Silk fibers in energy harvesting devices. a) Schematic of the core‐shell structure for the silk triboelectric yarns. Reproduced with permission.^[^ ^137^ ^]^ Copyright 2017, American Chemical Society. b) SEM image of a silk/stainless‐steel integrated yarn for TENG. c) Working principle of the yarn TENG for energy harvesting during stretching. d) Illustration of the application of silk energy harvesting devices. e) Picture and output signals of the TENGs integrated in a kneepad for movement monitoring. b–e) Reproduced with permission.^[^ ^28^ ^]^ Copyright 2020, Springer Nature.

Core‐sheath structures, in which conductive yarns and dielectric silk fibers function as the core and sheath layers, respectively, are developed in yarn TENGs.^[^ ^6^, ^136^ ^]^ As shown in **Figure** **15**a, the core–sheath silk yarn TENG can be further weaved or knitted into fabrics, which can be used to harvest energy from stretching or tapping with other materials.^[^ ^137^ ^]^ The silk/stainless steel yarn integrated with flexibility, conductivity, weaving, and triboelectric function is continuously produced by simple cowrapping spinning technology.^[^ ^28^ ^]^ The SEM image in Figure 15b shows a clear core–sheath structure of the silk‐wrapped TENG yarn. By further wrapping with the prestretched silicon rubber and another copper electrode, a stretchable energy harvester was developed, and the contact‐and‐separate of silk fibers and rubbers trigger the current in an external circuit and generate an instantaneous current, as seen from the illustration of working mechanism in Figure 15c.

Sandwiched structure^[^ ^119^, ^138^ ^]^ in which two conductive electrodes distributed in the outsides and single‐ or multilayer functional fibers are another well‐developed structure for energy harvesting devices. The sandwich structure piezoelectric nanogenerator (PENG) using spider silk exhibits a high energy conversion efficiency (≈66%), current (≈0.68 µA), high output voltage (≈21.3 V), and instantaneous power density of ≈4.56 µW cm^−2^. Electrospun silk nanofiber‐networked films with high surface‐to‐volume ratios are designed for the biotriboelectric generator. The bio‐TENG exhibits a high energy output due to the large surface area.^[^ ^45^ ^]^ Moreover, an all‐fiber hybrid piezoelectric‐enhanced friction nanogenerator is manufactured by electrospinning silk fibroin and polyvinylidene fluoride (PVDF) nanofibers between conductive fabrics. Owing to the large specific surface area of nanofibers and the extraordinary ability of silk fibroin to provide electrons during triboelectricity, the hybrid nanogenerator shows excellent electrical performance with a power density of 310 µW cm^−2^.^[^ ^139^ ^]^

To program the triboelectric property, recombinant spider silk proteins (RSSPs) for customizing the charge affinity were developed by genetically engineering technology.^[^ ^140^ ^]^ The inherent uniform chain length of RSSP improves the reliability and repeatability of mass manufacturing and has excellent transparency for parallel optical readout during energy harvesting. Inject printing and water lithography were utilized in the large‐scale production of a rough pattern on RSSP surfaces for the silk based energy harvesting device design.^[^ ^140^ ^]^ The silk fiber‐based wearable energy harvester exhibits good flexibility, air permeability, bendability, and weavability, which provides a new green method for sustainable, portable energy harvesting, and self‐powered physiological sensing (Figure 15d,e). More importantly, the biocompatibility of the SF facilitates a wider in vivo applicability of the energy devices.

## 5 Concluding Remarks

In this review, the strategy from mesoscopic functionalization of SF to smart, flexible silk fiber devices, and their applications in textile electronics and photonics are detailed.

We conclude that as an ancient natural textiles and human friendly material, the understandings on the meso‐structures and the correlation to the functionality will lay down the foundation to design and construct a new class of functional mesofibrous materials. These new meso materials can be further fabricated into fiber electronics/photonics. The hierarchical network structures of silkworm silk fibers are divided into five levels, namely, amino acid sequence, *β*‐sheet, and *α*‐helice. *β*‐crystallites, crystal network (nanofibrils), and nanofibril networks. These meso hierarchical structures play a key role in determining the macroscopic performances of silk fibers. In particular, since the crystallization force arising from *β*‐crystallization is generally strong, the meso hierarchical structures associated with crystal networks are relatively tough and stable. This gives rise to the toughness and durability of silk fibers, and the stable meso‐functionalization via molecules or nanoseeds templating nucleation. Namely, three main approaches for mesoscopic functionalization, i.e., meso‐reconstruction, meso‐doping, and meso‐hybridization can be identified. In this manner, nanoparticles with different functions are applied as nanoseeds to form meso‐functionalized silk fibers, enabling the original silk fibers with additional performance, i.e., mechanical, electrical, and/or optical functionalities. Based on this materials engineering principle, numerous functional components (molecular and nanoseeds) can be adopted to fabricate novel SF hybrid fibers that have new functionalities while maintaining the original performance. In this regard, nanofunctional seeds act as heterogeneous nucleation templates to promote SF nucleation that enables the adjustment of the mesoscopic hierarchical structures and the doping of functional components into the meso structures. Therefore, the functionalized SF fibers exhibit robust strength, good toughness, with additional functionalities, such as electrical conductive and magnetic, cellular, or fluorescence properties. In terms of various fiber spinning techniques, unfolded SF molecules in solutions can be transformed into full flexible and wearable skin friendly electronic or photonic fibers and textiles .

Evidently, meso‐functionalized silk fibers are successfully obtained in wearable sensing, movable energy harvesting, remote diagnosis, personalized medical healthcare, human–machine interaction, etc. Actually, mesoscopic engineering also provides an excellent strategy for the development of other functionalized silk fibers, i.e., magnetic fibers, self‐adjusting fibers, and self‐cooling fibers. Therefore, future research should focus on the multifunctionalization by mesoscopic structure engineering. Besides, researches in textile engineering should focus on the methods of integrating silk electronics/photonic devices into 2D or 3D fabrics by weaving, knitting, or braiding technologies without sacrificing the fiber functionalities and textile comfort, breathability, and flexibility.

This review will act as a key reference for the preparation of high‐performance macrofunctional RSF fibers via functionalization of the silk meso‐structure. Meso‐functionalized silk fibers are expected to become a crucial component of biocompatible wearable electronics, significantly driving our lives into a new era of internet‐integrated textiles.

## Conflict of Interest

The authors declare no conflict of interest.
